# The Influence of Tertiary Education Disciplines on Self-Construals and Conflict Management Tendencies

**DOI:** 10.3389/fpsyg.2021.659301

**Published:** 2021-06-04

**Authors:** Sheila X. R. Wee, Wan Yee Choo, Chi-Ying Cheng

**Affiliations:** School of Social Sciences, Singapore Management University, Singapore, Singapore

**Keywords:** self-construal, tertiary education, conflict management style, United States, Singapore

## Abstract

While cultural difference on self-construal are well-documented, how acculturation to a new cultural environment could change an individual’s self-construal remains under-explored. In this research, how tertiary education disciplines could be associated with the endorsement of self-construals which, in turn, affect students’ conflict management tendencies were explored. Study 1 revealed that across the United States and Singapore, college students from business and social science disciplines exhibited the trend of endorsing more independent and interdependent self-construal respectively, regardless of the different dominant self-construals in the two countries. Study 2 explored how tertiary education disciplines is associated with individuals’ conflict management tendencies via the endorsement of different self-construals among Singaporeans. Findings showed that individuals from business discipline possess a more independent self-construal and in turn endorsed more of a competing conflict management style than those from social sciences. Different disciplinary cultures could link to conflict management tendencies via the endorsement of self-construals, yielding significant theoretical and practical implications.

## Introduction

The concept of “self” is the foundation for all meaningful human behavior (Baumeister, 1999). For a long time, the “self” is defined as “the individual’s belief about himself or herself, including the person’s attributes and who and what the self is.” This is believed to be true for all people across cultures and countries. It is not until three decades ago, Markus and Kitayama (1991) identified that the conceptualization of self can be highly influenced by culture. According to Markus and Kitayama (1991), there are two types of self-construal, namely the independent and interdependent self-construal. Self-construal patterns are afforded by different cultures and thus vary across cultures where the independent self-construal dominates in Western societies while the interdependent self-construal dominates in most non-Western societies (Markus and Kitayama, 2010). The variations in self-construal patterns account for the differences across many observed behaviors (Markus and Conner, 2013).

As a result of increasing globalization and cultural mixingprocesses, individuals’ self-construal patterns can no longer be succinctly categorized by their cultural background. For example, many Asian societies experienced rapid modernization, chiefly driven by Westernization over the short span of a few decades and such progresses are accompanied by increased exposure to individualistic values and practices (Gelfand et al., 2011; Zeng and Greenfield, 2015; Ogihara, 2017). As a result, individuals in Asian countries might not be solely dominated by interdependent self-construal. Instead, the swing between interdependent and independent self-construals develops as a new trend in Westernized Asian societies such as Singapore (Ng and Lai, 2010; Kumar, 2013). It is imperative to examine dual self-construal considering the wide-ranging influence that self-construal has on societal outcomes from altruism (Dogan and Tiltay, 2020), environmental behavior (Dogan and Ozmen, 2019), conflict management (Oetzel, 1998), and subjective well-being (Cheng et al., 2011).

While past research evidenced that processes like Westernization may facilitate the endorsement of an independent self-construal in collectivistic societies (Cheng et al., 2011), no studies have yet examined the specific contextual factors that are involved in cultivating dual self-construal. As part of the modernization argument, researchers have contested that tertiary education is a key agent of transformation for young adults’ sense of self (Markus and Kitayama, 1991, 2010). In this research, we investigate the effects of tertiary education disciplines on the change of Asian students’ dominant interdependent self-construals. First, we contest that the tertiary education and the enrollment into specific academic disciplines foster the endorsement of one’s self-construal repertoire from the predominant independent and interdependent self-construals in North American and Asian context, respectively. This is so as the *culture* within different tertiary education disciplines may facilitate the endorsement of the independent self-construal to varying extent. Specifically, we assert that students enrolled in business will endorse an independent self-construal more than those enrolled in social sciences, which then impact on their endorsement of a competing style as their conflict management tendencies. We will elaborate this proposition in the ensuing sections.

### What Is Self-Construal?

Self-construal, a term first coined by Markus and Kitayama (1991) refers to how an individual defines the self with respect to their social relationships. While psychologists had long assumed that the self was only perceived as an autonomous and individualized unit (Geertz, 1974), findings from cross-cultural research (e.g., Shweder and Bourne, 1982; Triandis, 1989; Markus and Kitayama, 1991; Fiske et al., 1998) yielded evidence of significant differences between cultures that prompted further examination of the relationship between culture and the self. To consolidate the findings from cross-cultural research, Markus and Kitayama (1991) proposed the construct of self-construal and identified two main types of self-construal, namely the independent and interdependent self-construal.

The independent self-construal is defined as the view of the self as a single, individualized entity that is separate from others (Markus and Kitayama, 1991). When thinking about the self, individuals with well-developed independent self-construal are likely to reflect on their own abilities, traits, and attributes (Markus and Kitayama, 1991; Singelis, 1994). They are motivated to set themselves apart from others by highlighting their own uniqueness (Cross and Madson, 1997). The independent self-construal tends to dominate in Western societies as the presence of the individualistic culture nurtures individuals to construe the self as a distinct and independent entity. On the other hand, the interdependent self-construal is conceptualized as the view of the self as being interconnected with others around them (Markus and Kitayama, 1991). When thinking of the self, individuals with a well-developed interdependent self-construal are likely to reflect on their relationships and roles. For the interdependent selves, they aim to fit in with their social environment, are constantly adjusting their behaviors according to the social contexts, and tend to prioritize group goals over individual’s desires (Cross and Madson, 1997). The interdependent self-construal tends to dominate in non-Western societies where the collectivistic culture’s emphasis on interconnectedness between the self and others (Hofstede, 1980; Triandis, 1983) guides one to perceive others as an essential component of the self.

### The Construct of Self-Construal

While self-construal may vary across culture (Markus and Kitayama, 1991), several researchers argued that any given individual can also vary between the independent and interdependent selves (e.g., Trafimow et al., 1991; Singelis, 1994; Gudykunst et al., 1996; Dijksterhuis and van Knippenberg, 1998). Markus and Kitayama (1991, 2010) argued that cultural systems may shape individuals’ way of being independent or interdependent; therefore the cultural difference in self-construal will be more sound at the collective level than the individual level. That is, within any cultural context, individuals could differ in their internalization and resistance of cultural systems and demands (Kitayama et al., 2009; Gelfand et al., 2011). In comparison, Singelis (1994) suggested that both independent and interdependent self-construals can coexist within an individual, even though the culture one is exposed to may facilitate the development of one self-construal more strongly than the other (Triandis, 1989; Markus and Kitayama, 1991). In the measure of self-construal, Singelis (1994) conceptualized independent and interdependent as separate, independent, and orthogonal dimensions–one could be high (low) on independent and high (low) on interdependent self-construal.

To reconcile the very different conceptualization and measurement of self-construal, Vignoles et al. (2016) suggested that self-construal is better conceptualized as multidimensional, bipolar constructs at the individual level. In two studies with samples of over 55 cultural groups in 33 nations, Vignoles et al. (2016) proposed a seven multifaceted dimensions of self-construal that captures the ways that individuals can be independent or interdependent across seven domains of personal and social functioning. The seven domains include (a) how individuals define the self (i.e., difference vs. similarity), (b) experience the self (i.e., self-containment vs. connection to others), (c) make decisions (i.e., self-direction vs. receptiveness to influence), (d) look after oneself (i.e., self-reliance vs. dependence on others), (e) move between contexts (i.e., consistency vs. variability), (f) communicate with others (i.e., self-expression vs. harmony), and (g) deal with conflict of interests (i.e., self-interest vs. commitment to others). Each of the seven dimensions can be captured by a bipolar independent-interdependent way of being. Across dimensions, one’s self-construal may vary to reflect the endorsement of both self-construals at a given time (Singelis, 1994; Gudykunst et al., 1996; Oyserman et al., 2002; Vignoles et al., 2016). The recent reconceptualization and measurement model of self-construal has been shown to be more consistent with Markus and Kitayama’s (1991, 2010) theorization of cultural and individual difference in self-construal. Recent research has also shown that the use of different self-construal dimensions improved theoretical predictions of self-construal’s and self-efficacy (Smith et al., 2016) and motivation (Yang and Vignoles, 2020). Therefore, we follow Yang and Vignoles’ (2020) most recent theorization of self-construal as a bipolar multidimensional construct.

### Tertiary Education Institutions and Self-Construals

Cultural demands in different cultures have been shown to lead to the development of different self-construal (Hofstede, 1980; Triandis, 1983; Markus and Kitayama, 1994). An important agent of socialization includes the exposure to tertiary education institutions. Markus and Kitayama (1994, 2010) posited that institutions like schools play a fundamental role in shaping individuals’ sense of self. Specifically, the values and norms that are advocated by these institutions are often endorsed by their members and could exert an influence on how they define the self. Across the different stages in all education systems across cultures, we contend that tertiary education would be the most critical when it comes to examining the individual differences in endorsement of different self-construals.

Tertiary education often serves as a key transition phase to adulthood for many students where they experience greater autonomy and independence (e.g., Goldscheider and Davanzo, 1986). Upon entry into tertiary education institutions, individuals are exposed to an influx of values, alternative perspectives, and new knowledge which encourages individuals to embrace an individualized and self-chosen set of beliefs and values (Perry, 1970; Oketch et al., 2014). The experience of tertiary education is argued to be largely contingent on the discipline one is enrolled in to. To illustrate, a comparison between business disciplines (e.g., operations, strategy) and social science disciplines (e.g., psychology, sociology), would reveal differences in the nature of the knowledge and content being taught, methods of assessments, teaching styles and values. Such disciplinary differences can then influence the degree to which one’s experience of tertiary education may encourage the endorsement of different self-construals.

#### Culture of Academic Disciplines

To understand how tertiary education disciplines may impact the students’ endorsement of different self-construals, we investigate this phenomenon by taking on a cultural approach. Indeed, some researchers have proposed that academic disciplines may be best understood through an examination of its values (e.g., Becher and Trowler, 1989; Kennedy, 1997; Walvoord et al., 2000) or in other words, the discipline’s *culture*. Academic disciplines are argued to have developed their own distinctive culture, with persistent patterns of shared values, beliefs, and assumptions among individuals within that area of study (e.g., Kuh and Whitt, 1988; Becher and Trowler, 1989; Lee, 2007). Becher and Trowler (1989) suggests that academic disciplines resembled that of “tribes.” For members (e.g., faculty members, students) who belong to specific “tribes,” they develop day-to-day intellectual and social practices that are consistent with the shared expectations and norms of their disciplinary cultures (Becher and Trowler, 1989; Krause, 2014). Through conceptualizing discipline as a culture, we could then better clarify how these norms, values, and practices that are advocated within each discipline may subsequently influence how individuals perceive and make sense of the self.

In this research, we compare the effects of business disciplines and social science disciplines on the endorsement of self-construals among college-students. These two disciplines represent notable distinctive academic cultures that warrant further examination on their influences on individuals (e.g., Biglan, 1973a). This comparison would allow us to gather important insights into the different aspects of discipline cultures between business and social science and how it could possibly influence the self-construals of young college students who are enrolled in these respective disciplines.

#### Cultures of Tertiary Education Disciplines: Business and Social Science

The dominant values in business are agentic, dominant, achievement-oriented, ambitious, aggressive, and rational (Ibarra et al., 2013; Zheng et al., 2018). In line with the dominant values in business, previous studies investigating goal orientations found that faculty members from business tend to prioritize character development and intellectual self-actualization (e.g., shaping students to be creative-thinker, to articulate their independent thoughts) as key teaching goals (Smart and Elton, 1982). When evaluating students’ activities within business disciplines, the cultures within business schools are typically characterized by the pursuit of status and are generally more power-oriented (Mccabe and Trevino, 1995; Sagiv and Schwartz, 2000). Supporting this observation, business students were found to be more concerned with maximizing their own welfare which further underscore their competitiveness and independence from others (Brutus and Greguras, 2008; Luo et al., 2011). Students in the business discipline are also trained to articulate their independent opinions and make autonomous decisions (Knight and Jack, 1992; Jung and Shin, 2018)–fostering them to be independent from others.

In contrast, the disciplinary cultures in social sciences are often less competitive, more communal, and interdependent (Sagiv and Schwartz, 2000). To begin with, the nature of knowledge taught within social science disciplines is described as one that is more reiterative and holistic (Neumann, 2003). Across United States and Europe, social science discipline adopt the use of situational and social explanations (Elchardus and Spruyt, 2009) to explain and direct students attention toward inequality and socio-political issues (Räsänen and Wilska, 2007; Venetoklis and Räsänen, 2012). This has led to more egalitarian and pro-social attitudes toward others amongst social science (vs. business) students (Chatard and Selimbegovic, 2007). Moreover, Sagiv and Schwartz (2000) identified social science disciplines as more loosely structured with less competition and more person-oriented. Faculty peers in social sciences are more receptive to collaboration and multiple authorships, fostering a more collaborative and harmonious work environment (Biglan, 1973b; Neumann, 2003). Similarly, Sawyer (1966) also reported that social science students tend to accommodate to their in-group members (e.g., friends or peers) compared to business students. Compared to business students, students in the social sciences are also more likely to possess benevolent values (Sagiv and Schwartz, 2000) and act in accord with principles of exchange and reciprocity, extending help to those who have helped and supported them (Sawyer, 1966).

#### Tertiary Education Disciplines’ Endorsement of Self-Construal

The culture in business disciplines replicate that of a pro-independence environment where espoused values like competition, novelty, autonomy, and independence (e.g., Hofstede, 1980; Triandis, 1989; Schwartz and Bilsky, 1990; Brutus and Greguras, 2008) are highly salient and are endorsed by their members. Business disciplines are characterized as more power-oriented and competitive (Ibarra et al., 2013) where individuals prioritize their own goals above that of others (Brutus and Greguras, 2008). Exposure to such an environment may demand individuals to construe the self in highly independent terms and exhibit characteristics including assertiveness and tough-mindedness (Lounsbury et al., 2010). The culture within a business discipline would therefore foster students to be more self-interested. In comparison, social sciences discipline would foster an interdependent self-construal. The social science disciplinary culture is described as more person-oriented and accommodating as individuals look out for the needs of others around them (Sawyer, 1966). As such, individuals who are enrolled in social sciences disciplines are exposed to an environment that would pose a less strong demand for them to construe the self in highly independent terms. Hence, they are less compelled to endorse a strong sense of independent self-construal compared to their peers from business disciplines.

Even though different cultures exert different demands on individuals to construe the self as independent or interdependent (Markus and Kitayama, 1991, 2010), we contend that the enrollment into different academic discipline will influence changes on self-construal across cultures. In an individualistic society, enrollment into a business discipline would reinforce students’ independent self, while a social science discipline would breed an interdependent self. In a collectivistic society, the culture within a social science discipline would continue to reinforce students’ interdependent self, while the demands of a business discipline would foster an independent self.

We intend to capture the differences of self-construal endorsement in different academic discipline cultures in our research by following the common practice in the study of self-construals and operationalize both self-construals as a continuum (Vignoles et al., 2016). More specifically, we contend that academic disciplines will enact its influence on Vignoles et al.’s (2016) self-containment vs. connection to others dimension. The dimension of self-containment vs. connection depicts one’s experiencing of the self. Specifically, it contrasts a sense of self-containment (e.g., “I consider my happiness separate from the happiness of my friends and family”) with a sense of connection to others (e.g., “If a person hurts someone close to me, I feel personally hurt as well”). This dimension is found to be significantly associated with the value of individualism vs. collectivism such that higher individualism leads to higher self-containment and vice versa. It is also found to be associated with religious heritages such that self-containment is highest among Protestants, middle among Buddhists, and lowest among Muslims.

We argue that this dimension best reflect the demands of different academic disciplinary culture on students’ self-construal. To illustrate, the demands of a business discipline (e.g., character development, competitiveness) fosters students to be more self-contained by the values and norms of independence, assertiveness, self-reliance and personal achievement, which are very much equivalent to the pursuit of self-containment. As for exposure to a social science discipline, students are shaped to be interdependent, communal, egalitarian and reciprocal, which can be summarized as being more connected to others. Some might argue that the difference of the two disciplinary cultures could also be related to the two dimensions of self-reliance vs. dependence on others and self-interest vs. commitment to others of Vignoles et al.’s (2016) seven dimensions of self-construals. We argue against this notion because the training of social science might encourage students the values of egalitarian and concerns for the larger collective’s needs, it does not facilitate dependence^1^. In addition, whereas business majors facilitate students to pursue independence, competition and personal achievement, it should not be qualified as pure self-interest as self-content and achievement can help fulfill group’s needs and interests in many cases, such as CEO’s assertiveness can be translated to the company’s performance and merits. As Vignoles et al.’s (2016) dimensions are bipolar constructs, a higher self-containment identification reflects an independent self-construal, and less identification with this independent way of being reflects a more interdependent self-construal. Consequently, we will use independent self-construal to refer to self-containment in our hypothesis testing.

H1: *Individuals from business disciplines are more likely to endorse a higher level of independent self-construal than individuals from social science disciplines.*

### Impact of Self-Construals on Conflict Resolution Tendencies

In social life, people cannot avoid social conflict. A conflict can be defined as a process in which one perceives that their own interests are being opposed by another party (e.g., Donohue and Kolt, 1992; Rahim and Magner, 1995). To deal with conflicts, individuals develop conflict management styles that depict their general tendencies or responses toward a conflict (Putnam and Poole, 1987; Ting-Toomey, 1997). Research shows that self-construal shapes important interpersonal differences in conflict management (e.g., Oetzel and Ting-Toomey, 2003; Utz, 2004). Considering that different academic cultures could give rise to different self-construals, it suggests that students with different academic training would approach interpersonal situations, such as conflict in a drastically different manner.

### Conflict Management Style

Conflict management style is defined as one’s general tendencies or responses toward a conflict (Putnam and Poole, 1987; Sternberg and Dobson, 1987; Ting-Toomey, 1997). Rahim (1983) identified five types of conflict management styles that emerged from two basic dimensions of concern for the self and concern for the others (Rahim and Bonoma, 1979). Concern for the self captures the degree (high or low) to which an individual is motivated to satisfy their own needs and concerns while concern for others measures the degree (high or low) to which an individual is motivated to fulfill or satisfy the concerns of the other person. The five conflict are: (a) avoiding style (low level of concern for self and others) associated with withdrawal, (b) obliging style (low level of concern for self and high level of concern for others) associated with satisfying the concerns of the other party at the expense of individual’s needs, (c) dominating style (high level of concern for self and low level of concern for others) associated with looking to win their own position and ignoring the needs or concerns of the other party, (d) integrating style (high level of concern for self and others) associated with attempting to resolve the problem with an effective solution that satisfies both parties’ needs, and (e) comprising style (intermediate in concern for self and others) associated with give-and-take to achieve a mutually acceptable decision. To simplify the categorization of the five styles into competitive and cooperative conflict management styles, dominating belongs to a competitive style whereas the rest belong to a cooperative style.

### Self-Construal and Conflict Management

Research on self-construal and communication revealed that self-construal can predict one’s conflict management style (e.g., Oetzel, 1998; Ting-Toomey et al., 2001; Utz, 2004). Individuals who are higher on independent self-construals are generally more competitive than those with a higher interdependent self-construal and tend to place less emphasis on preserving the other party’s image (Oetzel and Ting-Toomey, 2003). Research also shows that self-construal served as a better predictor for one’s conflict behaviors compared to demographic variables like one’s ethnicity or gender (Oetzel, 1998; Ting-Toomey et al., 2001).

Individuals with a high level of interdependent self-construal often prioritize the maintenance of harmonious relationships over the pursuit of personal goals (Markus and Kitayama, 1991; Cross and Madson, 1997). Furthermore, individuals who are higher on interdependent self-construal are more concerned about being well-liked by the other party and are more sensitive toward the needs and feelings of the other party (Kim, 1993). This suggests that interdependent self-construal may be associated with more cooperative conflict management styles. In contrast, individuals with a dominant independent self-construal are likely to place their individual needs and concerns first (Markus and Kitayama, 1991; Utz, 2004). They are generally more particular about the clarity in communication and prefer direct and explicit communication styles (Brown and Levinson, 1987). Hence, independent individuals are more likely to utilize competitive conflict styles like dominating compared to the interdependent selves. Together these suggests that individuals’ self-construal will be predictive of one’s conflict management style.

H2: *Independent self-construal (vs. interdependent self-construal) will be positively associated with competitive conflict management styles.*

H3: *Independent self-construal (vs. interdependent self-construal) will be negatively associated with cooperative conflict management styles.*

### Disciplines, Conflict Management Tendencies, and the Mediating Role of Self-Construal

Of utmost importance, the types of academic disciplines could possibly influence conflict management tendencies. As we have established, the disciplinary culture in business disciplines would represent one that is similar to that of an individualistic culture. Building on Markus and Kitayama’s (1991) argument concerning the interplay between culture and the self, we assert that individuals from business disciplines are more likely to endorse a stronger independent self-construal than individuals from social science disciplines. Hence, we expect individuals from business disciplines to display greater competitive conflict management styles than their peers from social science disciplines.

H4: *Individuals from business disciplines would display greater competitive conflict management styles compared to individuals from social science disciplines.*

H5: *Individuals from business disciplines would display lower cooperative conflict management styles compared to individuals from social science discipline.*

Bearing in mind the relationship between tertiary education discipline and the cultural demands for different self-construal, as well as the relationship between self-construal and conflict management styles, it suggests that the relationship between tertiary education discipline and conflict management style would be positively mediated by the endorsement of an independent self-construal. We propose that enrollment into a business discipline would positively lead to a preference for a competitive conflict management style that is mediated by an increased independent self-construal.

**FIGURE 1:**
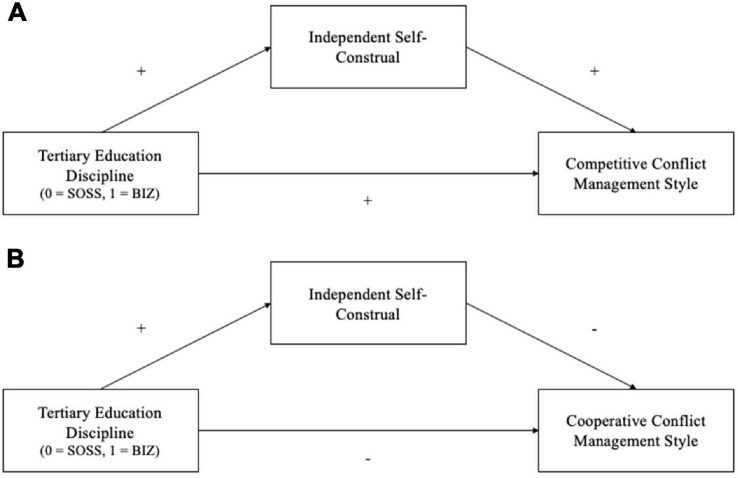
Hypothesized mediation models with independent self-construal as the mediator for tertiary education discipline and competitive conflict management style (H6) in panel **(A)**, and cooperative conflict management style (H7) in panel **(B)**. SOSS and BiZ refers to social science and business discipline respectively.

H6: *The relationship between types of disciplines and competitive conflict management styles will be positively mediated through one’s self-construal (Figure 1A).*

H7: *The relationship between types of disciplines and cooperative conflict management styles will be positively mediated through one’s self-construal (Figure 1B).*

## Study 1: Endorsement of Self-Construal Overtime

In Study 1, we examined the individual differences in the endorsement of self-construals, specifically the dimensions of self-containment vs. connection to others by investigating the effects of tertiary education disciplines among American and Singaporean college students. Individuals from business disciplines would endorse a higher level of independent self-construal on the dimension of self-containment vs. connection to other compared to their peers from social science disciplines (H1). This is because the culture within business disciplines strongly encourage self-containment and would demand students to acquire an independent view of the self on the two self-construal dimensions. In comparison, students in social science disciplines are compelled to pursue connection to the larger community. As such, there is less emphasis on self-containment that could aid in the development of the independent self-construal compared to that of business disciplines. While college students are intensively exposed to their academic discipline culture during their college years and are acculturated to a professional in their discipline, it is expected that the level of independent self-construal will differ across students from business and social science disciplines.

We contend that the effects of tertiary education discipline enrollment would result in different levels of demand for students within an individualistic and collectivistic culture. Americans are known to be high on individualism whereas Singaporeans are high on collectivism (Hofstede and Minkov, 2010). Consequently, an American culture affords an independent self-construal (Markus and Kitayama, 1991; Cross et al., 2010) while a Singaporean culture facilitates an interdependent self-construal (Kurman, 2001). By recruiting American and Singaporean college students with different dominant self-construals, we predict that the strength of influence of academic discipline cultures will influence college students’ self-construal to different extents.

## Methods

### Participants

One hundred and seventy-one Singaporean undergraduate students (136 female; *M*_*age*_ = 22.20, *SD* = 1.44) who majored in Business and Social Sciences were recruited from a Subject Pool System. Singapore undergraduate participants received either two course credits or $10 (∼US$7.07) in exchange for their participation. In addition, 230 American undergraduate students (157 female; *M*_*age*_ = 25.30, *SD* = 5.06) majoring in Business and Social Sciences were recruited using Qualtrics panel service. Participants were in their first (*n*_*biz*_ = 20 biz; *n*_*soss*_ = 6)^2^, second (*n*_*biz*_ = 81; *n*_*soss*_ = 43), third (*n*_*biz*_ = 78; *n*_*soss*_ = 37), and fourth year (*n*_*biz*_ = 101; *n*_*soss*_ = 35) of undergraduate study. American participants were recruited from the Qualtrics panel service and remunerated accordingly for their participation. Sample size conducted through G^∗^Power (Faul et al., 2007, 2009) was determined through a power analysis of 0.80 power to detect a significant culture × discipline interaction.

### Procedure

Participants were recruited through the Subject Pool System in a local Singapore university or through the Qualtrics Panel in the United States. The survey included a series of questions asking about their schooling years, disciplines, and self-construal patterns. Demographic information was collected at the end of the survey before participants were debriefed. All study data are available online at osf.io/t5qwh/.

### Measures

#### Self-Construal

Participants were tasked to report their self-construals on the 22-item Vignoles et al.’s (2016) Multi-dimensional Self-construal measure, with a focus on the two-item sub-dimension of self-containment vs. connection to others^3^. The items read as “Your happiness is unrelated to the happiness of your family (reversed coded),” and “If someone in your family is sad, you feel the sadness as if it were your own.” Ratings were made on a seven-point scale from strongly disagree (1) to strongly agree (7). Correlation of the two items is 0.240, *p* < 0.001^4^. Higher ratings on the measure indicate an independent self-construal while lower scores reflect an interdependent self-construal.

Demographic variables including schooling years, discipline, age and gender were collected at the end of the study and included as covariates for all analyses as they were found to be significantly correlated with our key variables (Table 1).

**TABLE 1:** Means, standard deviations, and correlation matrix for the variables in Study 1.

	***M***	***SD***	**Skewness**	**Kurtosis**	**1.**	**2.**	**3.**	**4.**	**5.**
1. Country	1.58	0.49	−0.32	−1.91	–				
2. Age	24.28	5.21	1.05	0.35	**0.34**	–			
3. Gender	1.73	0.45	−0.98	−0.81	−**0.12**	−**0.29**	–		
4. Discipline	0.70	0.46	0.88	−1.23	**0.14**	0.06	0.07	–	
5. Schooling Years	2.90	0.95	−0.26	−1.08	**0.11**	**0.25**	−0.07	0.05	–
6. Self-Containment vs. Connection to Others	3.52	1.17	−0.23	−0.03	−**0.26**	−0.04	−**0.15**	0.08	0.06

*Country (1 = Singapore, 2 = United States). Gender (1 = male, 2 = female). Discipline (0 = social sciences, 1 = business). Schooling years (1 = freshmen, 2 = sophomore, 3 = junior, 4 = senior). Boldface denotes *p* < 0.05.*

## Results

### Self-Construal, Discipline, and Culture

We first conducted an omnibus multiple regression model with discipline and culture as the predictors, self-containment vs. connection to others as the outcome variable, and age and gender as covariates. Results from the regression analyses revealed a main effect of discipline on self-construal, *B* = −1.06, *SE* = 0.39, *t*(400) = −2.75, *p* = 0.006. Consistent with H1, business students (*M* = 3.58, *SD* = 1.32) were more self-contained compared to students from the social science (*M* = 3.38, *SD* = 1.04) discipline, *F*(1, 400) = 8.20, *p* = 0.004, η_*p*_^2^ = 0.02. The main effects of culture on self-construal was also found to be significant, *B* = −1.31, *SE* = 0.34, *t*(400) = −3.88, *p* < 0.001. Singaporean undergraduate students (*M* = 3.87, *SD* = 0.86) were more self-contained compared to American undergraduate students (*M* = 3.26, *SD* = 1.29), *F*(1, 400) = 21.15, *p* < 0.001, η_*p*_^2^ = 0.05. Lastly, the interaction between discipline and culture significantly predicted for students’ independent self-construal, *B* = −0.48, *SE* = 0.24, *t*(400) = 1.97, *p* = 0.049, η_*p*_^2^ = 0.10. Planned contrast revealed that there was no cultural difference in self-containment between Singaporean (*M* = 3.50, *SD* = 0.87) and American (*M* = 3.24, *SD* = 1.20) social science students, *F*(1, 117) = 2.89, *p* = 0.092, η_*p*_^2^ = 0.02. However, business students from Singapore (*M* = 4.08, *SD* = 0.77) were significantly more self-contained compared to American business students (*M* = 3.26, *SD* = 1.31), *F*(1, 276) = 32.00, *p* < 0.001, η_*p*_^2^ = 0.10. The results from our omnibus analysis and planned contrast revealed that compared to students from the social science discipline, business students endorsed higher levels of independent self-construal, supporting H1.

To explore the potential endorsement of self-construal over time within different discipline, we conducted a multiple regression model with types of discipline (business vs. social sciences) and schooling years (freshmen vs. sophomores vs. juniors vs. seniors coded as 1, 2, 3, and 4) as predictors, while self-containment vs. connection to others was entered as the outcome variable. Culture, age, and gender was included in the model as covariates. Results from the multiple regression revealed that the main effects of discipline (*B* = 0.81, *SE* = 0.39, *t*(400) = 2.08, *p* = 0.039) and schooling year (*B* = 0.61, *SE* = 0.18, *t*(400) = 3.46, *p* = 0.001) were significant. Specifically, business (vs. social science) students (*M*_*biz*_ = 3.58, *M*_*soss*_ = 3.38), and senior (vs. freshmen) students (*M*_*seniors*_ = 3.57, *M*_*freshmen*_ = 2.88) were more self-contained. A significant interaction between discipline and schooling year emerged, (*B* = −0.40, *SE* = 0.13, *t*(400) = −3.08, *p* = 0.002), indicating that the effects of discipline on students’ self-construal depended on students’ exposure to their discipline culture at different levels of their program. Social science students were less self-contained over each school year (*M*_*freshmen*_ = 3.75, *M*_*seniors*_ = 2.97, *B* = −0.12, *SE* = 0.05, *t*(120) = −2.30, *p* = 0.023), while business students became more self-contained over each school year (*M*_*freshmen*_ = 2.63, *M*_*seniors*_ = 3.77, *B* = 0.23, *SE* = 0.07, *t*(279) = 3.17, *p* = 0.002).

## Discussion

Different from previous findings on culture and self-construals, American college students do not possess higher independent self-construals compared to Singaporean college students on self-containment vs. connection to others. This could be due to the fact that Singapore is a Westernized Asian country that used to be colonized by Britain and is currently composed of multicultural and multiracial groups (Clammer, 1982; Chua, 2003). Most importantly, our findings revealed that students enrolled in the business discipline endorsed a higher level of independent self-construal on the self-containment vs. connection to others dimension compared to their peers from the social science discipline supporting H1. Our additional analyses showed that academic training at different levels of the program reflect a more independent self-construal. At the end of their 4 year training, business students endorsed a stronger independent self-construal compared to social science students across both American and Singaporean college students.

Findings in Study 1 provide initial evidence for the tertiary academic discipline’s impact on students’ self-construal both in the United States and Singapore. Our results therefore provide nuance to previous research showing that enrollment into tertiary education fosters endorsement of a stronger independent sense of self (Oketch et al., 2014). Indeed, the demands within the business and social science disciplines nurtured the endorsement of a more independent and interdependent self in students across time and culture, respectively. The results from Study 1 showed that academic disciplines are associated with different dominant self-construal and can potentially nurture different self-construals via a disciplinary acculturation process. In Study 2, we examine the relationship between academic discipline, self-construal, and conflict management style within a Singapore.

## Study 2: Mediating Role of Self-Construal Between Academic Discipline and Conflict Management Style

As seasoned students from the business discipline endorsed a higher level of independent self-construal compared to their peers from the social science discipline (H1), it suggests that tertiary education and the types of discipline that students are enrolled in impacts students’ self-construal endorsement at different levels. Previous research has shown that self-construal has a direct and significant impact on one’s conflict management style (e.g., Oetzel, 1998; Oetzel and Ting-Toomey, 2003). This suggests that academic discipline could nurture the preference for different conflict management styles through the endorsement of different self-construals.

In Study 2, we investigate the mediating relationship between academic discipline and conflict management style through self-construal in Singapore. We predict that the endorsement of a higher level of independent self-construal would lead to a preference for more competitive conflict management style (H2). While the endorsement of a higher level of interdependent self-construal would lead to a preference for more cooperative conflict management style (H3). We predict that students in business disciplines would display more competitive conflict behaviors compared to students in social science disciplines (H4) and less cooperative conflict behaviors (H5) as they would endorse a higher level of independent self-construal. Lastly, the relationship between tertiary education discipline and conflict management tendencies is positively mediated by self-construal (H6 and H7).

## Methods

### Participants and Procedure

One hundred and seventy-one undergraduate participants were recruited in a local Singapore University. A *post hoc* power analyses conducted using G^∗^Power (Faul et al., 2007, 2009) revealed that the statistical power to detect a moderate effect size (*f*^2^ = 0.25) was more than adequate, 0.90. Participants were recruited from a Subject Pool System and received either two course credits or $10 (∼US$7.07) in exchange for their participation (136 females; *M*_*age*_ = 22.20, *SD* = 1.44). As participants were in their second (44.4%), third (33.3%), and fourth year (22.2%) of their undergraduate studies, all participants have had at least a year of schooling experience within their disciplines. Among the 171 participants, 37.4% were from social sciences disciplines and 62.6% were from business disciplines.

Importantly, several participants had declared a second major from either business or social science schools (e.g., Marketing and Psychology, Sociology, and Operations). There were 27 (42.2% of social science discipline participants) participants from social sciences disciplines with a second major in business, and eight (7.5% of business discipline participants) participants from business disciplines with a second major in social sciences. Results from our independent *t*-tests comparing these groups revealed no significant differences on our variables of interest. Hence, we proceeded with our analyses by classifying the participants based on their first majors only (i.e., business disciplines or social science disciplines).

Participants were asked to complete an online survey within 2–3 days after signup. The survey included a series of questions asking about their self-construal patterns, conflict management tendencies, and other general behaviors. Demographic information was collected at the end of the survey before participants were debriefed. All study data are available online at osf.io/t5qwh/.

### Materials

#### Self-Construal

To measure dimensions of self-construal, we administered Vignoles et al.’s (2016) Multi-dimensional Self-Construal measure used in Study 1.

#### Conflict Management Tendencies

To measure one’s conflict management tendencies, we utilized Rahim’s Organizational Conflict Inventory-II (Rahim, 1983) which consists of 28 items on a five-point Likert scale from strongly disagree (1) to strongly agree (5).

Demographic variables including schooling year, discipline, age, and gender were collected at the end of the study (Table 2). We similarly controlled for age and gender in our following analyses as they were found to be significantly associated with our variables of interests (Table 3).

**TABLE 2:** Descriptive statistics for the variables in Study 2.

	***M***	***SD***	**Skewness**	**Kurtosis**	**Reliability**
1. Age	22.17	1.50	0.76	0.89	–
2. Gender	1.79	0.41	–1.43	0.05	–
3. Discipline	0.63	0.49	–0.52	–1.75	–
4. Schooling Years	2.77	0.79	0.44	–1.27	–
5. Self-Containment vs. Connection to Others	3.83	0.85	0.21	0.90	**0.203**^*a*^
6. Dominating	3.17	0.72	–0.17	–0.23	0.770
7. Obliging	3.53	0.58	–0.32	0.05	0.795
8. Integrating	4.15	0.41	–0.16	1.18	0.813
9. Avoiding	3.40	0.68	–0.45	–0.03	0.794
10. Compromising	4.01	0,52	–0.60	1.63	0.745

*Gender (1 = male, 2 = female). Discipline (0 = social sciences, 1 = business). Schooling years (2 = sophomore, 3 = junior, 4 = senior). Self-containment refers to self-containment vs. connection to others. Boldface denotes *p* < 0.01.*

*^*a*^The self-containment vs. connection to others scale is composed of only two items, therefore we reported the inter-item correlations in place of the scale’s Cronbach’s alpha (Pallant, 2011).*

**TABLE 3:** Correlation matrix for the variables in Study 2.

	**1.**	**2.**	**3.**	**4.**	**5.**	**6.**	**7.**	**8.**	**9.**
1. Age	–								
2. Gender	−**0.55**	–							
3. Discipline	0.10	−0.00	–						
4. Schooling Years	**0.45**	0.03	0.12	–					
5. Self-Containment vs. Connection to Others	**0.18**	−**0.20**	**0.33**	0.03	–				
6. Dominating	−0.01	−0.11	0.05	0.12	**0.24**	–			
7. Obliging	−0.10	**0.16**	−0.12	−0.03	−0.13	−0.06	–		
8. Integrating	−0.01	0.06	−**0.16**	0.03	−0.13	−0.01	0.02	–	
9. Avoiding	−0.07	**0.18**	0.04	−0.05	−0.09	−0.11	**0.54**	−0.03	–
10. Compromising	−0.08	**0.20**	−**0.22**	−0.03	−0.13	−0.05	**0.33**	**0.55**	**0.17**

*Boldface denotes *p* < 0.05.*

## Results

### Types of Disciplines and Self-Construal

We first analyzed if there were differences in self-construal patterns across types of disciplines within the Singapore undergraduate student. An ANCOVA was conducted with type of discipline (business vs. social sciences) as the independent variable, and self-containment vs. connection with others as the outcome variable, while controlling for our age and gender. Results revealed a significant effect of disciplines on one’s self-containment, *F*(1, 170) = 20.13, *p* < 0.001, η_*p*_^2^ = 0.11. As hypothesized in H1, seasoned students belonging to business discipline reported being more self-contained (*M*_*biz*_ = 4.08, *SD* = 0.77) than students from social science discipline (*M*_*soss*_ = 3.50, *SD* = 0.87).

### Self-Construal and Conflict Management Tendencies

To analyze the relationship between self-construal and conflict management tendencies, we conducted several regression analyses with self-containment vs. connection to others as the independent variable, and each competitive conflict management style as the outcome variable.

With respect to competitive conflict management tendencies, regression analysis revealed a significant positive effect of self-containment vs. connection to others on the choice of a dominating strategy, *B* = 0.21, *SE* = 0.07, *t*(170) = 2.98, *p* = 0.003, lending support for H2. Taken together, the results from our analysis showed that independent self-construal is positively related with competitive conflict tendencies like dominating (H2).

However, an examination into the relationship between self-construal and cooperative conflict management tendencies revealed that self-containment vs. connection to others did not predict cooperative conflict management styles such as obliging, integrating, avoiding, and compromising, *p* > 0.05. H3 was not supported.

### Types of Disciplines and Conflict Management Tendencies

An ANCOVA was conducted to identify if there are potential differences in conflict management tendencies across disciplines (H4). Types of disciplines was included as our independent variable and conflict management styles were included as dependent variables. Results revealed no significant differences between tertiary education disciplines in the use of dominating as a competitive conflict management style, *F*(1,170) = 0.53, *p* = 0.466, η_*p*_^2^ = 0.003. There was no difference in the use of dominating as a competitive conflict management style between students in the business (*M*_*biz*_ = 3.20, *SD* = 0.74) and social sciences (*M*_*soss*_ = 3.12, *SD* = 0.70) discipline.

When cooperative conflict management styles were included into ANCOVA models, we found a significant difference in the use of integrating (*F*(1, 170) = 4.79, *p* = 0.030, η_*p*_^2^ = 0.03) and compromising conflict tendencies (*F*(1, 170) = 8.91, *p* = 0.003, η_*p*_^2^ = 0.05). More specifically, business students compared to social science students, displayed lower integrative (*M*_*biz*_ = 4.10, *M*_*soss*_ = 4.24) and compromising (*M*_*biz*_ = 3.93, *M*_*soss*_ = 4.18) conflict tendencies. We did not find any difference in the types of discipline and students’ subsequent use of obliging and avoiding conflict management style, *p* > 0.05. The findings partially supports H5.

### Mediation Analysis

To test our proposed mediation models (see Figure 1), we used Hayes (2013) PROCESS Macro Model 4. Academic disciplines were entered as the predictor variable (0 = social sciences, 1 = business), conflict management styles as the dependent variables and independent self-construal as the mediator. With reference to Baron and Kenny (1986) recommendations for mediational analyses, the non-significant relation between self-construal (proposed mediator) and cooperative conflict tendencies suggests that the proposed mediation with cooperative conflict strategies (H7) would not be theoretically supported. Therefore, we focused our analysis on dominating competitive conflict management style. Although Baron and Kenny (1986) tests for mediation suggests that one should establish the direct effects between the independent variable and the dependent variable before we can test for a mediation, stimulation studies have shown that a significant indirect effect may still be present in the absence of a total or direct effect due to other unaccounted factors in the current analyses (Rucker et al., 2011).

**FIGURE 2:**
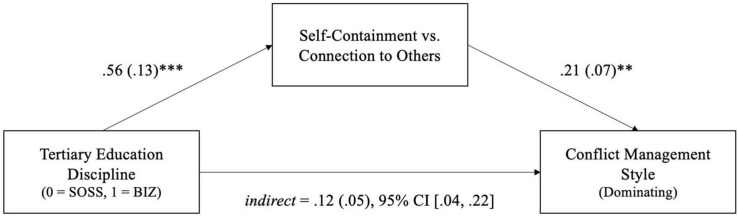
Mediating role of independent self-construal between discipline and competitive conflict management style mediation model with tertiary education discipline (0 = social sciences, 1 = business) as the predictor, self-construal as the mediator, and competitive conflict management style as the outcome variable. All values reflect standardized coefficients and standard errors in parentheses. **p < 0.01; ***p < 0.001.

Our mediational analysis revealed a significant mediation effect of self-containment vs. connection to others for the relationship between discipline types and dominating conflict management style. Individuals from business disciplines were found to report higher level of self-containment, β = 0.56, *SE* = 0.13, *t*(167) = 4.49, *p* < 0.001, and self-containment was positively associated with the use of competitive conflict management tendencies like dominating, β = 0.21, *SE* = 0.07, *t*(166) = 2.98, *p* = 0.003. Although we found a non-significant direct effect of discipline on dominating conflict management style, β_*direct*_ = −0.03, *SE*_*Boot*_ = 0.12, *t*(166) = −0.27, *p* = 0.788, 95%CI_*Boot*_ [−0.27, 0.20], the indirect effects of discipline on dominating conflict management style via self-containment was found to be significant, β_*indirect*_ = 0.12, *SE*_*Boot*_ = 0.05, 95%CI_*Boot*_ [0.04, 0.22], reflecting a full indirect-only mediation (see Figure 2 of Zhao et al., 2010) that supports H6 (Figure 2).

## Discussion

Our results from Study 2 provided further support for the relationship between types of tertiary education disciplines and self-construal (H1) exemplified in Study 1. Study 2’s results revealed and replicated the relationship between independent self-construal and competitive conflict management tendencies (H2, H4). Most importantly, our results revealed that the relationship between tertiary education discipline and competitive conflict management styles was significantly and positively mediated by one’s endorsement of independent self-construal (H6). Individuals from the business disciplines reported a higher endorsement of independent self-construal which accounts for their greater usage of dominating conflict management styles compared to their peers from social science disciplines. This demonstrates the importance of tertiary education disciplines in shaping important psychological outcomes like how individuals construe the self which, in turn, allows us to better identify and anticipate the types of conflict management styles one may utilize. Although a longitudinal study would provide a more convincing arguing for tertiary education discipline’s impact on self-construal over time, our current cross-sectional findings provide promising preliminary evidence illuminating the difference between business and social science discipline’s impact on self-construal and conflict management at different schooling year. Taken together, this lends support to our assertion that tertiary education disciplines such as business and social science majors would impact the endorsement of independent self-construal which then result in differences in the display of competitive conflict behaviors among Singaporeans with different educational background.

However, our findings did not yield support for the relationship between types of tertiary education disciplines, self-construal, and cooperative conflict management style (i.e., no support for H3 and H7; partial support for H5). Although individuals from business disciplines did report a lower tendency to utilize cooperative conflict management styles like integrating and compromising than peers from social sciences disciplines (H5). This did not hold for obliging and avoiding styles. Self-construal was not associated with the use of these cooperative conflict strategies and could not explain the discipline difference in cooperative conflict tendencies (H7).

This could be due to the unique impact of the various cultural influences on Singaporeans who are enrolled in tertiary education institutions. As we have posited, the disciplinary culture within social sciences is considered to be more interdependent and communal, hence, we would expect individuals from social science disciplines to exhibit more cooperative conflict management tendencies like obliging and avoiding. However, we only noted significant differences in the use of integrating and compromising styles but not obliging and avoiding, which emphasizes that individuals from social science disciplines do not entirely forgo their own interests at the expense of satisfying the other party’s. Rather, it appeared that they considered the other party’s interest alongside their own’s and attempted to satisfy both. Research into cultural differences in conflict management styles has shown that collectivistic and individualistic individuals alike often perceive integrating and compromising as the same conflict management strategy (Cai and Fink, 2002). Specifically, the model posits integrating as one that reflects a high degree of concern for the self and other, while compromising reflects an intermediate in concern for self and others. While this is not consistent with our hypothesis, it is in line with Rahim’s (1983) theoretical proposal of the dual-concern model where integrating is considered a “win-win” strategy. This demonstrates that when Singaporeans’ exposure to individualistic values and Western cultures within the society prompts them to endorse a certain degree of independent self-construal on top of their default interdependent self-construal, they do find a balance between the two and seek to satisfy and fulfill their own interests to a certain extent. However, one’s tertiary education discipline would, importantly, account for the observed individual differences in the acquisition of independent self-construal and thereby, conflict management tendencies.

## General Discussion

Our research explored the endorsement of a self-construal through tertiary education discipline and the subsequent effect on conflict management strategies. While it is argued that institutions like schools have their own distinctive cultures, little is known about how that would go on to impact one’s self-construal. Through a cultural lens, we first demonstrated that enrollment into different academic discipline reflected different endorsement of independent self-construal across cultures. This provided first evidence that tertiary education offers a contextual and institutional influence for the endorsement of different self-construals.

Our findings demonstrated that tertiary education discipline was associated with the endorsement of independent self-construal across cultures. Americans and Singaporeans enrolled in a business discipline were more likely to endorse more independent self-construal. The endorsement of independent self-construal changed as a function of individuals’ academic discipline and schooling year. We showed that exposure to a business and social science discipline was associated with Americans’ self-construal endorsement across schooling year while exposure to different disciplinary culture showed more effect on Singaporean students’ self-construal endorsement merely through students’ enrollment into different discipline and schooling year. Together, our results point toward tertiary education discipline as a contextual agent in facilitating an independent self-construal amongst Singaporeans.

Our findings demonstrate that disciplinary culture does have an impact on one’s sense of self which hints at the importance of one’s experiences (e.g., schooling experience) in shaping one’s selfhood. Even though it is clear that nation’s culture may promote the development of a dominant self-construal over another, the results from present studies emphasize the importance of subcultures within institutions in exerting a simultaneous effect on one’s self-construal. Crucially, it suggests that one’s self-construal can be seen as malleable and susceptible to influences from various experiences.

The key theoretical contribution of this paper is in its efforts in extending to the existing literature on self-construal and conflict management tendencies by, first, introducing the concept of self-construals and then, examining it jointly with the influence of contextual factors like tertiary education disciplines. While past studies have primarily focused on self-construal and conflict management tendencies (e.g., Oetzel, 1998; Oetzel and Ting-Toomey, 2003), present paper demonstrates the potential malleability of one’s self-construal and investigated its impact on conflict management tendencies. Furthermore, we also examined the differences in self-construal both in the United States and Singapore by drawing attention to the influence of contextual and institutional factors like tertiary education disciplines in facilitating the endorsement of different self-construals which is something that is often overlooked by researchers in the field.

In a practical perspective, these findings are essential in shaping our understanding of how Singaporeans with different disciplinary training will then interact with one another. Indeed, for parties who are involved in a conflict and were both from business disciplines, we may expect them to behave in a highly competitive manner. This is especially common in workplace where young graduates from business education background may display a strong sense of competitiveness. However, for parties that were from social science disciplines, they are likely to respond to conflicts within the workplace in a very different manner and may have different priorities. For instance, they may strive to maintain harmony among their co-workers and display the tendency to accommodate to other parties in the conflict. In this case, the experience provided by different types of tertiary education disciplines could influence the dynamics of the interactions among individuals which can affect many important personal or organizational outcomes. These findings provide implications on team composition and organizational management where conflict management is the key for group performance. Crucially, this line of research also contributes to the understanding of informational diversity on workgroup outcomes. Previous studies suggest that informational diversity (i.e., differences in knowledge and perspectives) may benefit team performance, especially when its associated with innovation and complex problem solving (Williams and O’Reilly, 1998) or making of comprehensive strategic decisions (e.g., Mello and Ruckes, 2006). It has also been found to facilitate learning and accumulation of new knowledge and skills (Ackerman and Humphreys, 1990). Finally, many of these young graduates would be entrusted with key leadership roles in the future. They will take over important roles in the society and their decisions and behaviors can have severe societal impacts. As such, having the knowledge of how these individuals may deal with conflicts would be imperative when it comes to selection of leader and leadership development.

### Limitations and Future Directions

A limitation of our study would be that we are unable to pin-down the causal link between tertiary education discipline and the development of independent self-construal. This is so as both Study 1 and 2 were correlational studies where participants were asked to report this self-construal and conflict management tendencies. Lacking a full experimental design risks us to a possibility of selection effects. In other words, it is possible that individuals with a higher independent self-construal may have chosen to enroll in business disciplines than social science disciplines. In this case, we are unable to discern the effects of disciplinary cultures on the acquisition of independent self-construal. A longitudinal study would allow researchers to examine the causal relationship between tertiary education discipline and the development of self-construal over time. As such, we recommend for future researchers to utilize longitudinal studies to investigate this phenomenon and better clarify the links between tertiary education disciplines and the development of self-construals. Future research could adopt a longitudinal design to examine the changes in self-construal patterns across time, which can lend further support to the causal relationship between tertiary education disciplines and self-construal patterns not only in Singapore but across different cultures.

Our study also serves as an initial comparison of how different types of tertiary education disciplines can influence the endorsement of self-construals and subsequently, exert an impact on conflict management tendencies. Our findings provide a promising avenue for researchers who are keen to explore the interplay between education, self-construal, and psychological outcomes like conflict resolution. Future studies may consider examining a wider range of disciplines (e.g., engineering) to better discern the effects of disciplinary cultures on one’s perception of the self. Interestingly, some studies that explored the concept of gender-professional identities also demonstrated how females belonging in STEM fields may experience conflicts between their gender and professional identities. In light of the present findings, it would be interesting and of great significance for future studies to examine the disciplinary cultures within STEM fields and how it may exert an influence on individual’s self-construal and consider how social roles like gender may moderate its effect on the acquisition of dual self-construals. For instance, some studies have shown that females tend to endorse a stronger relational interdependence self-construal (e.g., Markus and Oyserman, 1989; Cross et al., 2010) where they are more communally oriented compared to males. Therefore, it is possible that females in STEM fields may take longer to find a good balance between the two self-construals which could implicate their outcomes at work.

Finally, future research could also consider how tertiary education disciplines influences self-construal across different cultural bases of self-construal. While our findings from Study 1 illustrated the tertiary education discipline influenced students’ self-construal across United States and Singapore, tertiary education discipline seemed to have more effect on Singaporean students within a collectivistic (vs. individualistic) culture. Specifically, we demonstrated the proposed relations between business academic discipline, enhanced independent self-construal, and competing conflict management tendencies in comparison to social sciences discipline within Singapore in Study 2. However, an individualistic culture with a default independent self-construal population may effect different conflict management tendencies. Future research could explore how social science discipline may lead to a more cooperative conflict management strategy via the development of a more interdependent self over time.

## Conclusion

On the whole, present paper established that the individual differences in self-construal patterns differ according to one’s experience in tertiary education in cross-cultural settings. Specifically, within the Singaporean society, the tertiary education discipline one is enrolled in could importantly impact the way they construe the self which, in turn, explains their behaviors when dealing with conflicts. Our findings provided an important starting point to understand the contextual factors of self-construal development and the conflict management tendencies of future leader selection and leadership development upon graduation.

## Data Availability Statement

The datasets presented in this study can be found here: osf.io/t5qwh/.

## Ethics Statement

The studies involving human participants were reviewed and approved by Singapore Management University IRB. The patients/participants provided their written informed consent to participate in this study.

## Author Contributions

All authors designed the research. WC and SW performed the research and analyzed the data. SW and C-YC wrote the manuscript.

## Conflict of Interest

The authors declare that the research was conducted in the absence of any commercial or financial relationships that could be construed as a potential conflict of interest.

## Footnotes

1 The dimensions of self-reliance vs. dependence on others (*r* = −0.052) and self-interest vs. commitment to others (*r* = 0.000) were not significantly related to disciplinary cultures.

2 Singapore undergraduate students can only declare their major after their first year of study, therefore the recruitment of SOSS students in Singapore was limited to second to fourth year undergraduate students with a declared major in social sciences.

3 Although the subdimensions of similar to others vs. difference, self-direction vs. receptiveness to influence by others, and self-expression vs. harmony are the most closely related to cultural differences, additional analyses revealed that they were unrelated to academic discipline (Vignoles et al., 2016).

4 As Cronbach’s alpha values are sensitive to the length of a scale, inter-item correlations are a better representative of the scale reliability (Pallant, 2011). Given that the self-containment vs. connection to others scale is composed of only two items, the scale reliability is calculated by Pearson’s R as a split-half reliability instead of Cronbach’s alpha, following the suggestions of Pallant (2011, p. 97). The use of a scale with low reliability is prone to increased Type II error rates (Grewal et al., 2004). Type II error refers to the failure to detect significant effects when it is present. Through a series of Monte Carlo simulations, Grewal et al. (2004) have shown that Type II error rates decreases with the increase of sample size. Our power analyses revealed that our sample size of 400 in Study 1 and 171 in Study 2 were more than adequate in detecting a moderate effect size in the subsequent analyses.
